# IsomiR_Window: a system for analyzing small-RNA-seq data in an integrative and user-friendly manner

**DOI:** 10.1186/s12859-021-03955-6

**Published:** 2021-02-01

**Authors:** Ana M. Vasconcelos, Maria Beatriz Carmo, Beatriz Ferreira, Inês Viegas, Margarida Gama-Carvalho, António Ferreira, Andreia J. Amaral

**Affiliations:** 1 Lasige – Faculdade de Ciências, Universidade de Lisboa, Lisbon, Portugal; 2 BioISI - Biosystems & Integrative Sciences Institute, University of Lisboa, Faculty of Sciences, Lisbon, Portugal; 3 CIISA - Centro de Investigação Interdisciplinar Em Sanidade Animal, Faculdade de Medicina Veterinária, Universidade de Lisboa, Lisbon, Portugal

**Keywords:** IsomiR, miRNA, Annotation analysis, Functional analysis, Browser application

## Abstract

**Background:**

IsomiRs are miRNA variants that vary in length and/or sequence when compared to their canonical forms. These variants display differences in length and/or sequence, including additions or deletions of one or more nucleotides (nts) at the 5′ and/or 3′ end, internal editings or untemplated 3′ end additions. Most available tools for small RNA-seq data analysis do not allow the identification of isomiRs and often require advanced knowledge of bioinformatics. To overcome this, we have developed IsomiR Window, a platform that supports the systematic identification, quantification and functional exploration of isomiR expression in small RNA-seq datasets, accessible to users with no computational skills.

**Methods:**

IsomiR Window enables the discovery of isomiRs and identification of all annotated non-coding RNAs in RNA-seq datasets from animals and plants. It comprises two main components: the IsomiR Window pipeline for data processing; and the IsomiR Window Browser interface. It integrates over ten third-party softwares for the analysis of small-RNA-seq data and holds a new algorithm that allows the detection of all possible types of isomiRs. These include 3′ and 5′end isomiRs, 3′ end tailings, isomiRs with single nucleotide polymorphisms (SNPs) or potential RNA editings, as well as all possible fuzzy combinations. IsomiR Window includes all required databases for analysis and annotation, and is freely distributed as a Linux virtual machine, including all required software.

**Results:**

IsomiR Window processes several datasets in an automated manner, without restrictions of input file size. It generates high quality interactive figures and tables which can be exported into different formats. The performance of isomiR detection and quantification was assessed using simulated small-RNA-seq data. For correctly mapped reads, it identified different types of isomiRs with high confidence and 100% accuracy. The analysis of a small RNA-seq data from Basal Cell Carcinomas (BCCs) using isomiR Window confirmed that miR-183-5p is up-regulated in Nodular BCCs, but revealed that this effect was predominantly due to a novel 5′end variant. This variant displays a different seed region motif and 1756 isoform-exclusive mRNA targets that are significantly associated with disease pathways, underscoring the biological relevance of isomiR-focused analysis. IsomiR Window is available at https://isomir.fc.ul.pt/.

## Background

MicroRNAs (miRNAs) are small non-coding RNAs that derive from a precursor transcript, the pre-miRNA. This precursor forms a ~ 70 nt hairpin with two arms (5p and 3p), from which two functional miRNAs may derive. MicroRNAs play an important role as regulators of gene expression in eukaryotes. They are critical for the maintenance of normal physiology and serve as potential biomarkers and therapeutic targets for a wide spectrum of diseases [1]. The development of Next-Generation Sequencing (NGS) for small non-coding RNAs (small-RNA-seq) allowed the discovery of hundreds of novel human miRNAs, along with a fast paced expansion of the known miRNA repertoire encoded by a wide variety of animal and plant genomes. Moreover, the generation of a large number of small-RNA-seq datasets in recent years enabled the detection of miRNA variants. These include miRNAs with SNPs and isomiRs, variants to the canonical miR sequence that display additions or deletions of one or more nucleotides at the 5′ and/or 3′ ends. Additionally, they may display post-transcriptional editing of their internal sequence or untemplated nucleotide additions to the 3′ end (‘tailings’) [2]. Recent studies have revealed disrupted miRNA expression in different types of cancers, identifying them as relevant biomarkers [3]. Noticeably, these observations have been extended to isomiRs by a few recent reports [4, 5]. However, knowledge regarding isomiR function is still very limited and their biological relevance is not yet fully understood.

To enable the detailed investigation of isomiR types and abundance in small-RNA-seq datasets, both in clinical and research environments, the development of integrated and reliable pipelines is required. Pipelines should be user-friendly and run automated workflows to produce results efficiently, with minimal human technical errors, while ensuring reproducibility.

Most researchers analyze small-RNA-seq data following successive analysis steps, relying on specific and highly accurate software available for performing each step. These include adapter trimming [6, 7], sequence quality control [8], genome mapping [9, 10], read normalization and differential expression analysis [11], miRNA target prediction [12–14], novel miRNA prediction [15], and gene set enrichment analysis [16, 17]. The use of all these tools involves dealing with diverse sources of information, insufficient documentation, difficult installation procedures, and occasional incompatibility of software versions. Furthermore, it involves manually querying different database sources like miRBase [18], Ensembl [19], and RNAcentral [20], or having the skills to develop automated batch querying.

Within this context, several open-source tools have been published in recent years that propose pipelines for the systematic analysis of small-RNA-seq data, some of which include functionalities for the identification and quantification of isomiRs. We identified 13 tools in this category: miRMOD [21], CPSS [22, 23], DeAnnIso [24], isomiRex [25], miR-isomiRExp [26], mirSpring [27], IsomiRage [28], and isomiRID [29], Chimira [30], sRNA toolbox [31, 32], CBS-miRSEQ [33], CAP-miRSEQ [34] and UEA small RNA workbench [35] (Table 1). However, many of these tools have restrictions in input file size or require transfer of input files to online servers. These solutions are not compatible with real small-RNA-seq data, for which the input file for a single sample can easily exceed 12 Gb. Furthermore, many of these tools do not allow the analysis of several datasets and importantly, lack an adequate graphical user interface (GUI). This hampers the ability of researchers without bioinformatics skills to use them. Other limiting issues that were identified include: (a) failure to integrate different analysis modules; (b) lack of graphical reporting of results; (c) inability to identify other ncRNA species and all types of isomiRs and possible fuzzy combinations; and (d) lack of integration with analysis modules for inferring the functional impact of isomiRs. This clearly identifies the need for a novel tool for systematic, user-friendly exploration of isomiR expression and function.

**Table 1:** Comparison of functionalities of currently available isomiR analysis tools

	Multiple input files	Identification of sncRNAs	Types of IsomiRs	Novel miRNA	DE^*^	miRNA targets	Functional analysis	GUI
IsomiR Window	▪	▪##	f	▪	▪	▪	▪	▪
CPSS2.0	▪		a,b,c	▪	▪	▪	▪	▪
DeAnnIso			a,b,c		▪	▪	▪	▪
miR-isomiRExp			a,b,d		▪			
mirSpring			a,b		▪			
IsomiRage			a,b		▪			
isomiRID			a,b,c,d,e		▪			
miRMOD			a,b,d			▪		▪
Chimira	▪		a,b,c,d,e					▪
sRNAtoolbox	▪	▪	f	▪	▪	▪	▪	▪
CBS-miRSEQ	▪	▪	a,b	▪	▪	▪	▪	
CAP-miRSEQ	▪	▪		▪	▪			
UEA- Small RNA Workbench	▪	▪		▪	▪			▪

^*^Differential Expression

^a^3′end modifications; ^b^5′end modifications; ^c^internal editings; ^d^3′ tailings; ^e^internal SNPs; ^f^all types and fuzzy combinations of isomiRs

^##^Produces table with quantification of other types of snRNAs that can be used for differential expression via command line

Here we present the IsomiR Window system for integrated analysis of small-RNA-seq data, featuring automated processing of quality approved datasets. The system has a pipeline that uses well established tools for several steps of the analysis. This includes read mapping, miRNA prediction, miRNA and isomiR quantification, differential expression analysis and functional analysis. Additionally, IsomiR Windows integrates a new algorithm that allows the detection of all types of isomiRs, and their possible combinations. IsomiR Window can process several datasets and provides a user-friendly interface accessed through a Browser.

IsomiR Window is free and it is distributed as a virtual machine that contains all the required software, allowing it to be used on any workstation with any type of operating system. It further includes essential reference genomes, as well as the corresponding annotation databases (miRBase, RNAcentral and Ensembl), to enable the analysis of 21 different species of animals and plantas (see supported genomes in Table 2).

**Table 2:** Genome versions and gene annotation databases used and available through the IsomiR Window

Species name	Genome accession	Bioconductor DB
**Animal species**		
*Bos taurus*	bosTau9	org.Bt.eg.db
*Canis familiaris*	CanFam3.1	org.Cf.eg.db
*Capra hircus*	ARS1	Not available
*Danio rerio*	GRCz11	org.Dr.eg.db
*Drosophila melanogaster*	BDGP6	org.Dm.eg.db
*Equus caballus*	EquCab2	Not available
*Gallus gallus*	galGal6a	org.Gg.eg.db
*Homo sapiens*	GRCh38	org.Hs.eg.db
*Mus musculus*	GRCm38	org.Mm.eg.db
*Ovis aries*	Oar_v3.1	Not available
*Pan troglodytes*	Pan_tro_3.0	org.Pt.eg.db
*Rattus norvegicus*	Rnor_6.0	org.Rn.eg.db
*Sus scrofa*	Sscrofa11.1	org.Ss.eg.db
**Plant species**		
*Arabidopsis thaliana*	TAIR10	org.At.tair.db
*Brachypodium distachyon*	Brachypodium_distachyon_v3.0	Not available
*Oryza sativa Japonica Group*	IRGSP-1.0	Not available
*Solanum lycopersicum*	SL3.0	Not available
*Sorghum bicolor*	Sorghum_bicolor_NCBIv3	Not available
*Vitis vinifera*	12X	Not available
*Zea mays*	B73_RefGen_v4	Not available

IsomiR Window has two main components: a Perl pipeline and a Browser interface. The Perl pipeline processes the data and wraps all functionalities. The Browser interface provides a user-friendly way of defining analysis settings and starting and monitoring the automatic execution of the Perl pipeline. The Browser interface was designed to allow the analysis of several biological replicates using a maximum of two experimental conditions. The Perl pipeline can also be operated by invoking the pipeline scripts directly. This allows its application to a broader range of experimental designs, such as comparative genomic studies or time-course experiments. IsomiR Window further provides visualizations and results as tables and interactive graphs, which the user can customize. All results can be exported in PDF format or in multiple image formats. The analysis settings are saved in a report.

The Perl pipeline encodes a novel algorithm for the detection of isomiR species. This algorithm was validated with a controlled and simulated dataset, and shown to support the high confidence identification of isomiRs. To exemplify the use of the IsomiR Window pipeline through the Browser interface, we present the analysis of a large small-RNA-seq data set generated from Basal Cell Carcinomas (BCCs) [36]. The analysis revealed a previously unreported complex dynamics of isomiR expression for this dataset. In particular, it revealed the existence of differential isomiR expression between distinct types of BCCs. The differentially expressed isomiRs display a specific set of mRNA targets that are significantly associated with disease pathways, including *STAT5* and *NOTCH2*.

## Implementation

### Architecture and design

**Fig. 1:**
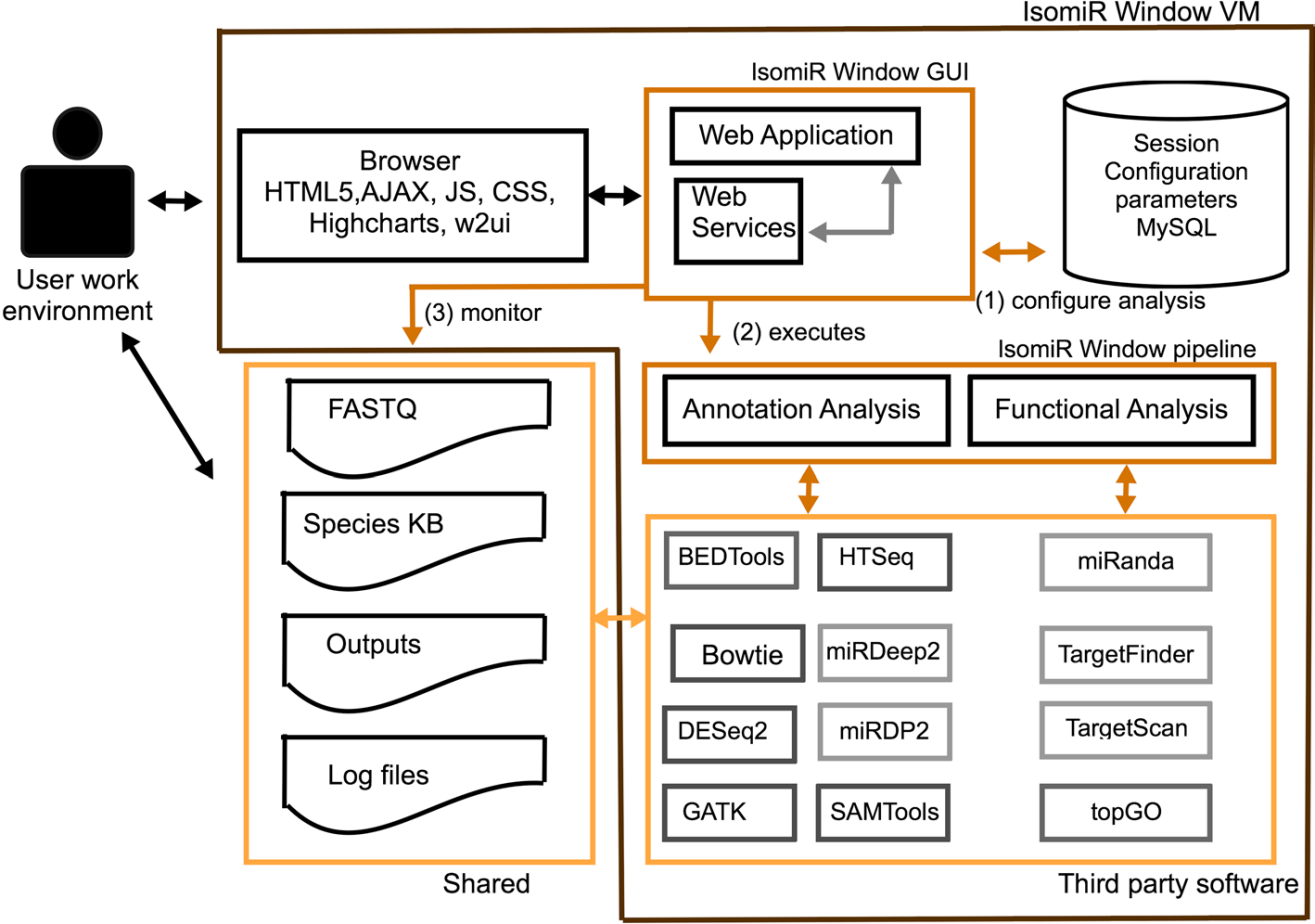
Integration between the IsomiR Window pipeline and IsomiR Window GUI. The GUI allows the user to visually set the analysis settings that are passed to the pipeline for execution. The pipeline is launched when requested by the user via de GUI and as the analysis is executed, log files are created by the pipeline. The GUI monitors these files in real time to present the user a progress meter. Gray boxes correspond to optional processes. Species KB stands for Species Knowledge Base that includes genomes and corresponding annotation

IsomiR Window (Fig. 1) has two layers of source code: (a) the IsomiR Window pipeline, written in Perl and R [37]; and (b) the IsomiR Window Browser interface, implemented using Laravel [38], an open-source PHP framework for simplifying Web application development. IsomiR Window is deployed in a virtual machine (VM). It can be run on ordinary workstations with any of the popular operating systems. The IsomiR Window VM was built on the Linux operating system (Ubuntu 18.04.3 LTS) and includes all required third party softwares. This allows users to circumvent frequent problems related with the difficulty of software installation. The VM interacts with a shared folder where input files, as well as files holding knowledge bases, are stored. This enables the faster download of the VM, and allows the user to only import the knowledge-base (KB) files for the species of interest.

The implementation of IsomiR Window was designed to provide two analysis modules (Annotation and Functional Analysis) that act at different functional levels. Each level represents an independent stage of the analysis. The tool was built considering a level-based design, allowing users to launch the workflows using all or some of the functionalities. This provides flexibility and optimizes efficiency when performing analysis using different parameters. The modular design enables a high degree of flexibility according to the user aims, while supporting the inclusion of additional functionalities and extensions in future versions.

### The IsomiR Window pipeline

The IsomiR Window pipeline facilitates the automated use of more than 10 open-source tools described in the literature that are well established for the analysis of small-RNA-seq data and sncRNA function (Fig. 1, Additional file 1: Table S1). Additionally, it incorporates a novel algorithm for the identification of all types of IsomiRs and all possible fuzzy combinations of sequence variations. This layer was developed using Perl and R code to perform data processing steps, including analysis and quality control steps to evaluate results. A shared folder between the user environment and the VM is created to store the input data and KB files. This strategy facilitates tool deployment and allows the user to store only the required KBs. The species KB comprise the species genome, using the most up-to-date assembly available (Table 2) and the corresponding annotation of ncRNAs (http://www.rnacentral.org), 5′UTRs (http://www.ensembl.org) and SNPs (https://www.ncbi.nlm.nih.gov/snp/).

### The IsomiR Window Browser interface

The Laravel framework [38] was used to design a Browser user interface with two modules of analysis that allow the information to flow between them. This structure makes the tool easily accessible to users with no bioinformatics skills. The Laravel framework supported the development of an interface with enforcement of data formats in form fields directly in the Browser. This is coupled to (1) a description of allowed values next to each field; (2) suggestions of default values; (3) messages that inform the user about the source of problems and possible solutions; and (4) contextual help, minimizing the chance of input errors by the user at the earliest possible stage, while offering a fluid work experience. The data is validated in the Browser using regular expressions in HTML attributes and JavaScript code for more complex cases. All data is double checked on the server side, taking advantage of Laravel controllers. A MySQL database is used to store the application data (users, configurations, experiments, etc.).

The Browser interface presents a form that allows the user to set analysis parameters in each of the analyses modules. Next, the user submits the form and the analysis begins. The commands to execute the Perl layer are automatically generated, feeding a series of background processes, which the user is able to monitor through the progress bar and by email. Finally, the results of the analysis are displayed in tables and charts.

### Simulated datasets and performance evaluation

**Fig. 2:**
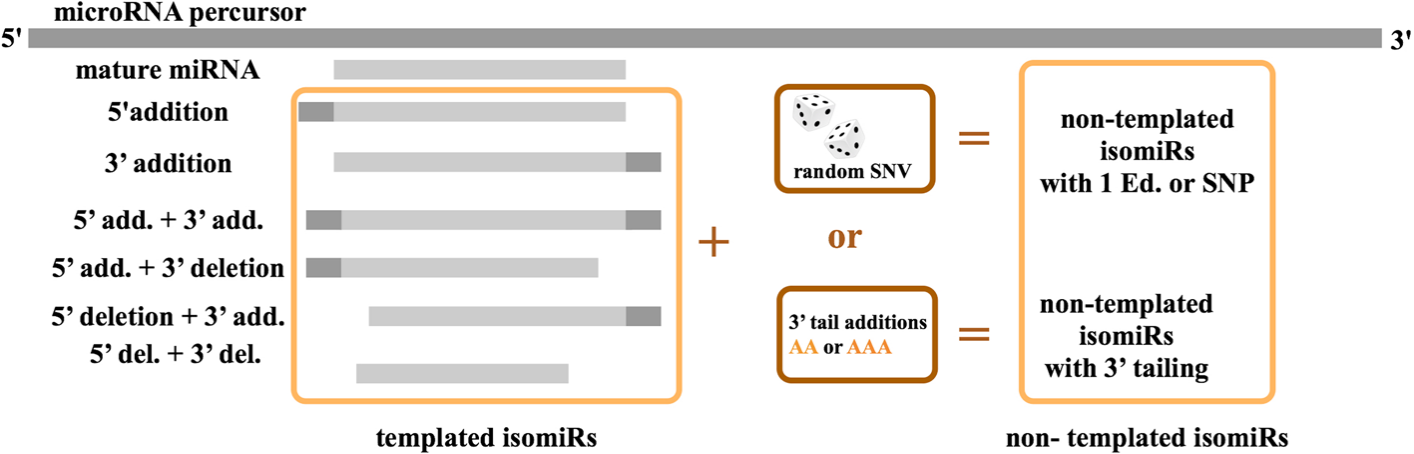
Schematic workflow for the simulation of small-RNA-seq datasets

Simulated 22-nt and 16-nt small-RNA-seq datasets were generated with ART [39] using miRBase v22.1 [40] hairpin and mature sequences of 22-nt-miRNAs (the most frequent size for human miRNAs) as templates. Average read depth was set to 1000 for hairpins and 10,000 for mature derived transcripts. These simulated sets were processed for mapping and selection of reads with zero mismatches. After mapping, using in-house developed Perl scripts, single nucleotide variants (SNVs) were inserted at random positions in reads, as well as 3′ tailing additions (Fig. 2). Results were classified as true positives (TP), false positives (FP) and false negatives (FN). Reads assigned to the same miRNA of origin were considered as TP. Reads assigned to other miRNAs than the one of origin were considered FP. Reads missing assignment were considered FN. Finally, we estimated the sensitivity (TP/(TP + FN)) and specificity (TP/(TP + FP)) to detect isomiRs with 3′end and/or 5′end offsets, combined or not with potential internal editing, SNPs and 3′end tailing.

## Reference datasets

We have tested the application and accuracy of IsomiR Window using 30 publicly available small-RNA-seq datasets from SRA study PRJNA148721 (SRR364267… SRR364296), previously used to investigate the role of miRNAs in Basal Cell Carcinomas (BCCs) [36]. This allowed us to demonstrate the application of IsomiR Window in human sequencing data, while addressing the role of isomiRs in BCCs. Raw data was adapter trimmed using Flexbar [6] and reads with 18–28 nts and an average quality score higher than 20 were selected for further analysis using Prinseq [41].

### IsomiR Window availability

The IsomiR Window VM and source code can be downloaded at http://isomir.fc.ul.pt. The Perl pipeline is available in separate as well at https://github.com/andreiaamaral/IsomiR-Window/.

## Results and discussion

We present here the IsomiR Window, a novel tool for the analysis of small-RNA-seq data aiming to detect, quantify and investigate the function of isomiRs. Our application consists of a pipeline that provides automated workflows, using well established tools for different analysis steps (mapping, miRNA prediction, miRNA quantification, differential expression analysis and functional analysis). IsomiR Window further incorporates a novel algorithm for the detection of all types of isomiRs and possible fuzzy combinations of corresponding sequence variations. IsomiR Window allows the processing of several datasets in parallel with no restriction of file size (restrictions are defined by the computational power of the workstation or server to be used). It further provides a user-friendly interface accessed through a Browser, producing results in an efficient timeframe, and minimizing human technical errors. This ensures reproducibility, supporting its use both in research and clinical settings.

### Workflow

The IsomiR Window tool is divided in two analytical modules that may be executed independently to analyze small-RNA-seq data. The analysis settings have been selected based on developer’s recommendations and on our daily experience in analysis of small-RNA-seq datasets.

**Fig. 3:**
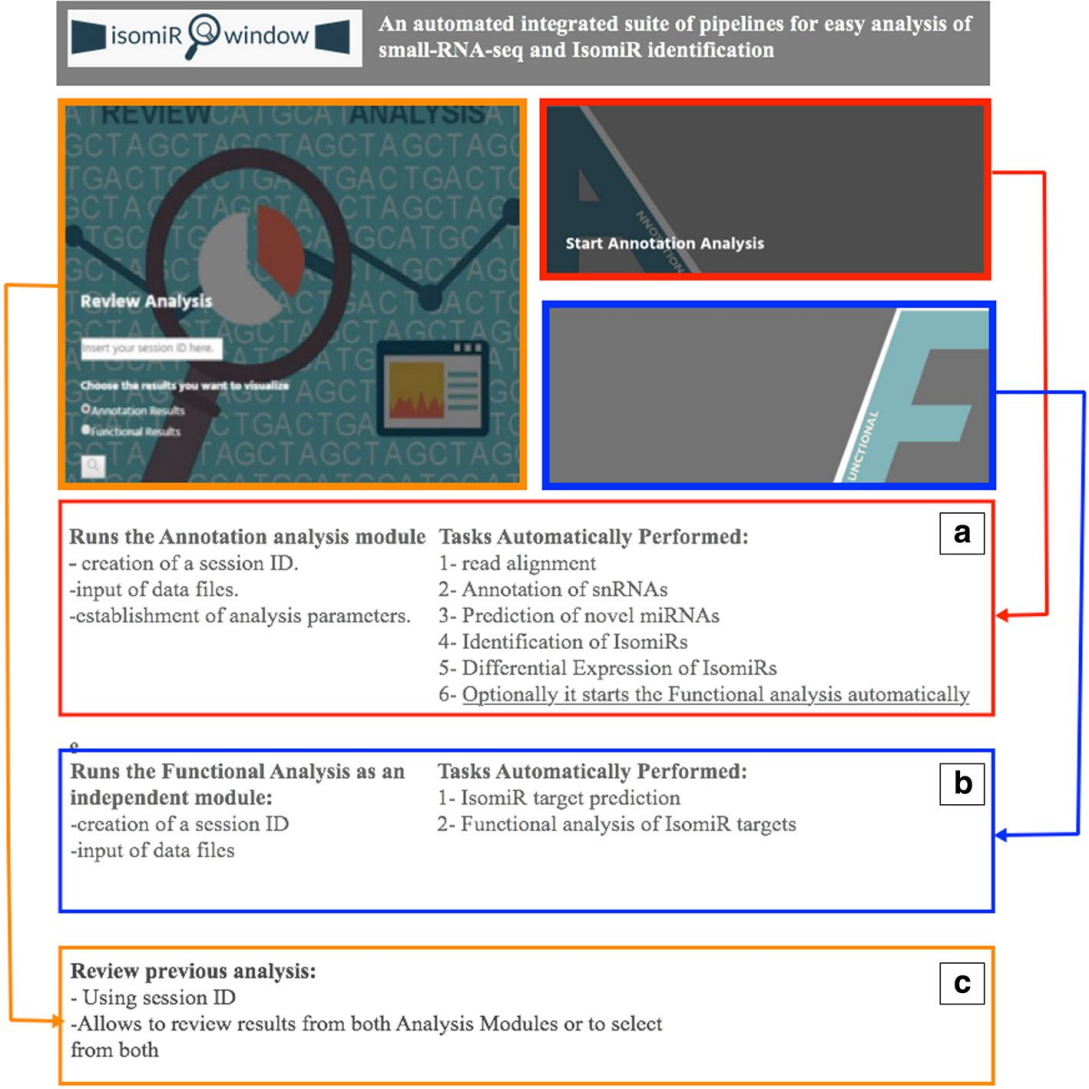
IsomiR Window homepage. Users may easily run all the analyses supported by IsomiR Window through its main interface. The interface provides users three different Modules: **a** The Annotation module that may automatically trigger the Functional analysis module; **b** the Functional analysis module that can be used independently of the previous; **c** review analysis that allows visualizing the results of a previous job. The interface provides users with easy to fill in forms allowing to set analysis parameters and to select to run in parallel different tools that perform the same analysis (e.g. isomiR target prediction) for posterior comparison

Our application automates the analysis steps, reducing human error and improving the reproducibility of results using this type of data. To this aim, IsomiR Window provides a GUI, through a Browser interface. The home page provides the user with the possibility to choose the type of analysis to run by selecting the desired analysis module (either Annotation analysis or Functional analysis—Figs. 1 and 3). The user may alternatively select the option to review the results of a previous analysis. After user selection, the Browser interface launches the IsomiR Window pipeline with the user-defined settings and executes the pipeline (Fig. 3), creating a session ID that will enable the user to review the performed analysis. The IsomiR Window pipeline was further conceived to perform a quality control check of input files. All the processes are monitored through log files to trace progress and errors in both data files and data analysis steps (Fig. 3). The complete results of IsomiR Window are saved in a project directory tree that maintains a structured organization for outputs files (Additional file 2: Fig. S1). The user can further export selected figures and tables in different formats (JPG, PNG, PDF) through the Browser. A report that includes the parameters used for the analysis is also produced in PDF format. IsomiR Window employs standard formats of input and output files, supporting experiments with paired and unpaired samples.

### Annotation analysis module

**Fig. 4:**
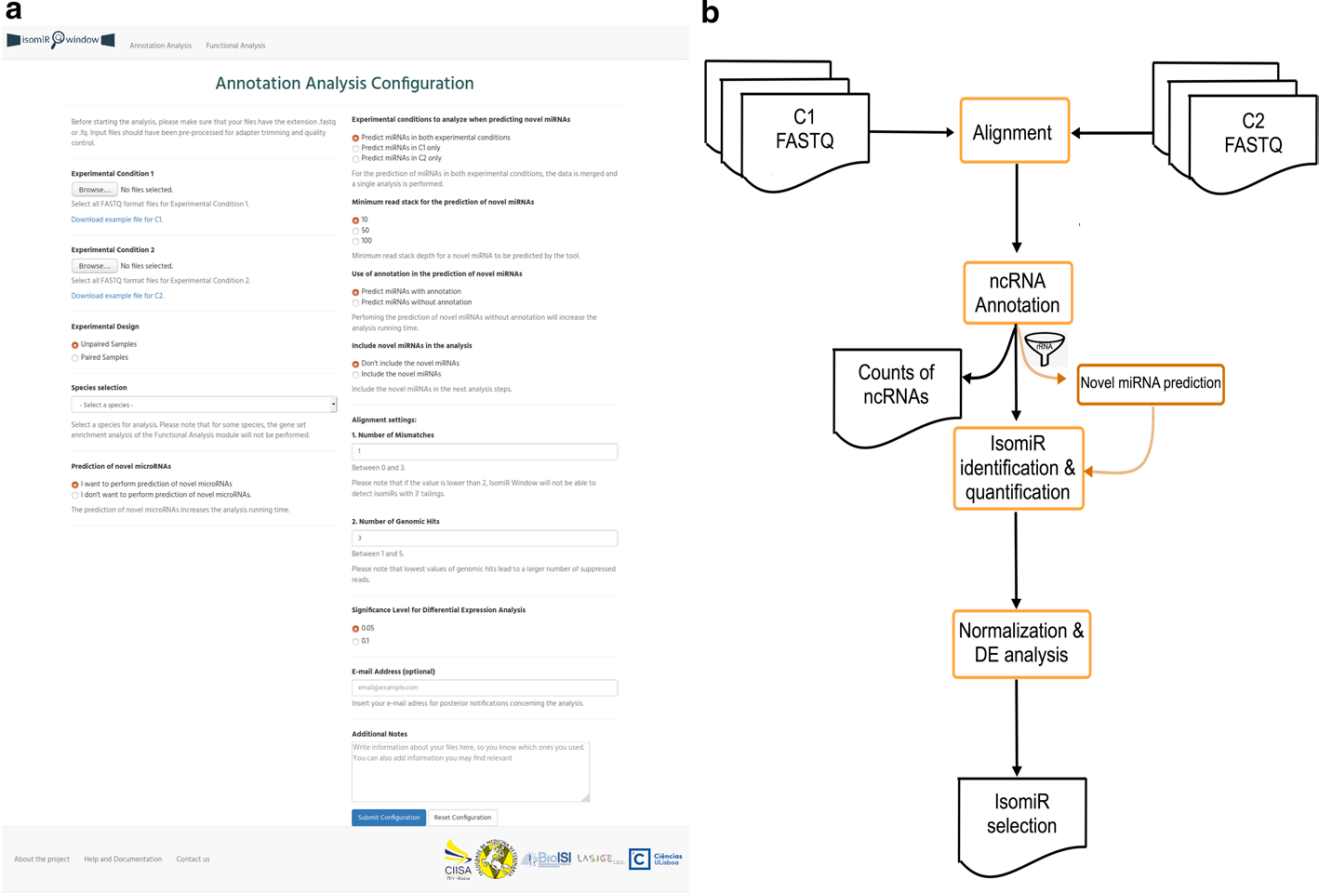
Annotation analysis module. **a** Display of the configuration analysis form. **b** Module workflow

Once the user selects the Annotation analysis module, the Browser is redirected to a form (Fig. 4a) where the user must select the input files for experimental condition 1 and experimental condition 2. This form allows the selection of several input files per experimental condition, with no limit on the number of biological replicates. The format of input files must be FASTQ. These FASTQ files should have been previously processed for adapter trimming and quality filtering. Then other parameters must be defined by the user: (a) experimental design (unpaired vs paired samples); (b) species; (c) prediction of novel miRNAs (optional due to longer run times) and possibility to include novel miRNAs in downstream analysis; (d) number of mismatches for read alignment (maximum of three, in order to support detection of isomiRs with 3′ end tailings); (e) number of genomic hits (maximum number of mapping possibilities for a read, ranging from one to five); and (f) significance level (desired level of statistical confidence for differential expression analysis, once the detection and quantification steps are completed). The form also contains an optional field for the user to insert an e-mail address to which the IsomiR Window will send updates regarding analysis progress. After filling in the form, the user must submit it by pressing the submit button. The IsomiR Window Browser interface will then produce a file holding the configuration of the analysis and will launch the IsomiR Window pipeline (Fig. 4b). The main steps of the workflow of the Annotation analysis module (Fig. 4b) are described in the following sections.

*1. Format control of input files*

In this step the Perl pipeline tests if the input corresponds to a FASTQ format.

*2. Short-read alignment*

This step uses the settings of number of mismatches and multiple hits defined by the user in the Annotation analysis form (Fig. 4a). The analysis is executed using *Bowtie* [10] in *best strata* setting. This allows an overall higher accuracy of short read mapping in comparison with other setting and other read mappers [42]. The obtained output is a SAM file. At the end of this step the Perl pipeline tests if it was concluded with success.

*3. Annotation of ncRNAs*

In this step, the SAM file is processed using HTSeq [43] to filter out reads corresponding to rRNA (by comparison of read mapping coordinates with annotation database coordinates), producing a Filtered_SAM* file. This tool also quantifies ncRNAs, producing a table of read counts for each of the identified species and storing it in the ncRNAs folder (Additional file 2: Fig. S1).

*4. Novel miR prediction*

If the user selected the option to perform the identification of potential novel miRNAs, the pipeline will run miRDeep2 [15] (in the case of animal data) or miRDeep-P2 [44] (in the case of plant data). This will produce information regarding sequence, mapping coordinates and statistical significance of novel miR candidates as an output. As miRNA predictors often predict overlapping miRNA precursors, in case of an overlap of at least 90%, the algorithm selects only the prediction with the highest coverage of reads. The analysis is run with the miRNA annotation from miRbase as input, using genomic coordinates of the latest assembly (Table 2) available from RNA Central. In case the user has chosen the option of adding the identified novel miRNAs to the species annotation, subsequent steps of the pipeline will take these as input.

*5. Identification and quantification of isomiRs*

**Fig. 5:**
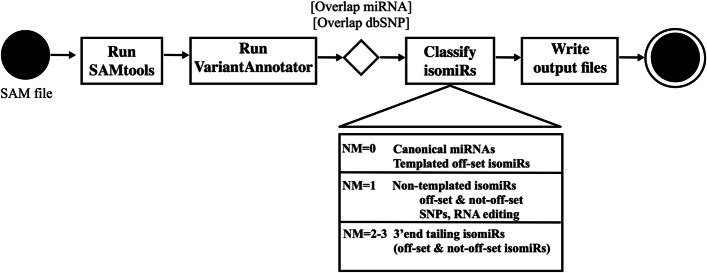
Activity diagram for find_isomiRs.pl. The left black circle represents the start of the activity, the right black circle represents the end of this activity. Rectangles represent actions, whereas diamond shapes represent decisions

Using the filtered SAM file produced in step (3), our new encoded algorithm “find_isomiRs.pl” will be launched (Fig. 5). The algorithm performs the comparison of the mapping coordinates of reads with annotated miRNA precursors and canonical miRNAs and classifies them accordingly. A conditional rule is that it only considers reads with a maximum shift of 5 nts in relation to the genomic coordinates of the canonical miRNA. It first processes reads without mismatches, followed by reads with a single mismatch, which potentially harbor internal editings. SAMtools [45] is used to identify true potential variants (-q20 –Q20) and BCFtools [46] is used to filter *bona fide* SNPs (-d10 -a3). Identified variants are then annotated using the GATK function VariantAnnotator [47] and classified as dbSNP if they occur in the dbSNP database [48]. Otherwise, in the case of animals, A to G transitions are classified as canonical A-to-I RNA editing [49]. In the case of plants, since dbSNP information is not available, RNA editing sites are not reported. Other types of variants are classified as potential new SNPs. Position shifts in comparison to the canonical miRNA location are estimated for both perfect match and one mismatched reads. Finally, the algorithm processes reads with 2 or 3 mismatches located at the 3′end. These reads potentially correspond to isomiRs with 3′end tailing. At the end of this step, the algorithm identifies all the reported types of isomiRs and their possible combinations. The output is a count table in which each line holds the isomiR_ID code, followed by the total number of reads in each biological replicate of each experimental condition. This file is saved by the IsomiR Window pipeline in the not_ambiguous folder (Additional file 2: Fig. S1). IsomiRs that display homology to more than one miRNA precursor are classified as ambiguous and not taken into account for the statistics, and are stored in the ambiguous folder (Additional file 2: Fig. S1).

**Fig. 6:**
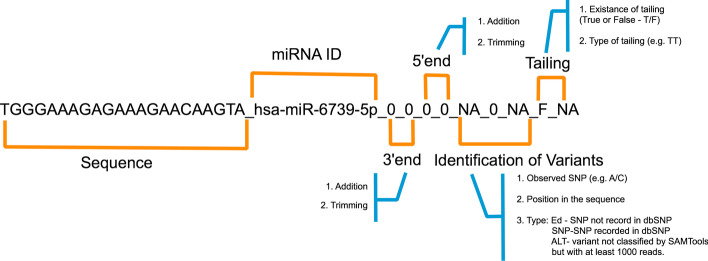
The IsomiR identifier. The figure depicts the identifier generated by the find_isomiR algorithm for a canonical miRNA. This identifier was developed in order to allow different queries and to generate summary statistics. The identifier should be read from left to right and displays the transcript sequence, the identifier of the canonical miRNA from which it derives, followed by a code that distinguishes the different types of frame-shifts encountered in comparison with the respective canonical miRNAs. Alterations (ALT) are variants not considered by samtools but that were observed in at least 1000 reads

The IsomiR_ID code (Fig. 6) is composed of 11 information fields linked by an underscore. It begins with the sequence of the transcript followed by the ID of the corresponding canonical miRNA. The next segment of the code is composed of four numeric fields that report the number of nucleotides in each type of possible off-sets. This is followed by three fields reporting on internal sequence variations (presence of potential SNPs or editing) and finally two fields describing the presence and type of 3′end tailing. This identifier allows the algorithm to efficiently estimate statistics regarding the abundance of the different types of isomiRs. Like the example in the figure, canonical miRNAs are also quantified and display shifts at the 3′end and at the 5′end equal to zero.

**Fig. 7:**
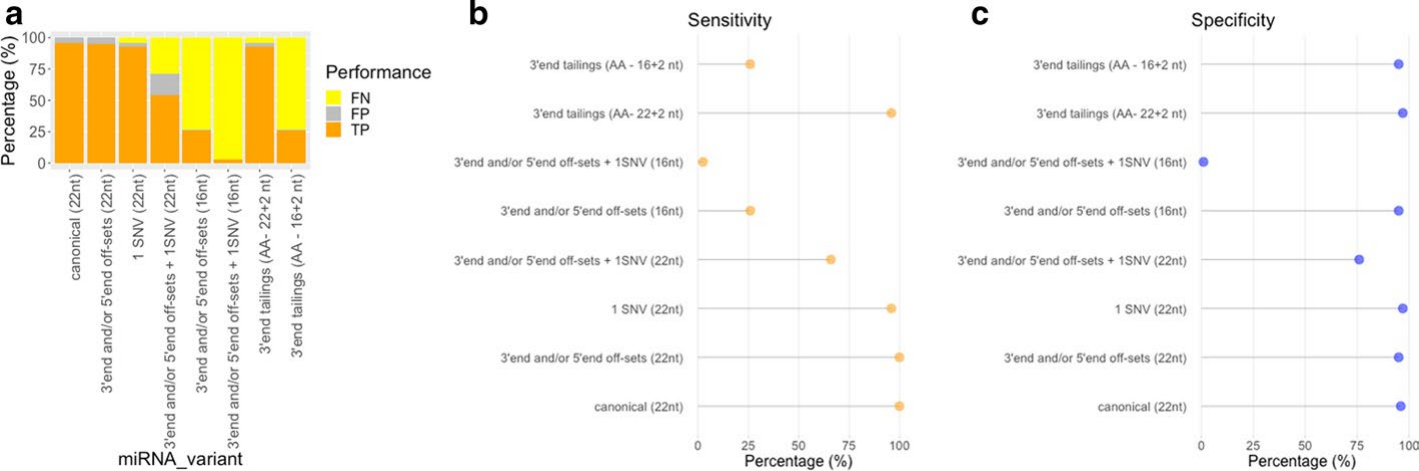
Performance testing of the find_IsomiRs.pl algorithm. Small-RNA-seq data was simulated in order to achieve higher number of transcripts in regions overlapping canonical miRNA sequences and simulating different read length and isomiR types. **a** Plot of the true positive, false positive and false negative values for different types of miRNA variants (canonical miRNAs and isomiRs). **b** Estimated sensitivity and **c** specificity values

Performance testing analysis showed that canonical miRNAs led to a small percentage of detected false positives (4–5%) (Fig. 7a). These false positives corresponded to reads incorrectly mapped by Bowtie to the genomic coordinates of a distinct miRNA. The percentage of false positives increased in off-set isomiRs, variants that vary from the canonical miRNA at the 3′end or 5′end (22nt). These results are similar to those obtained with the isomiRID algorithm [29], which also depends on Bowtie for correct positional assignment of miRNA variants. In the case of isomiRs with one SNV, the rate of false negatives increased. Many of these variants do not reach enough depth to be called as such by samtools [45], given the fact that find_isomiR.pl has encoded stringent criteria for variant calling in order to minimize the percentage of false positives. The percentage of false negatives reached higher values in isomiRs with 16nts and one SNV. This low sensitivity is expected since these isomiRs are both poorly mapped and their sequencing depth is too low for reliable detection. IsomiRs with off-sets at the 3′end and/or the 5′end, SNVs and 3′end tailings were detected with similar levels of sensitivity and specificity as canonical miRNAs (Fig. 7b, c).

*6. Analysis of differential expression*

Using the read count table of isomiR expression, the pipeline invokes R and the DESeq package [11] to normalize counts and test for differential expression of miRNAs (including all possible variants and novel miRNAs if requested by user).

At this point the analysis automatically progresses to the Functional Analysis module.

### Functional analysis module

**Fig. 8:**
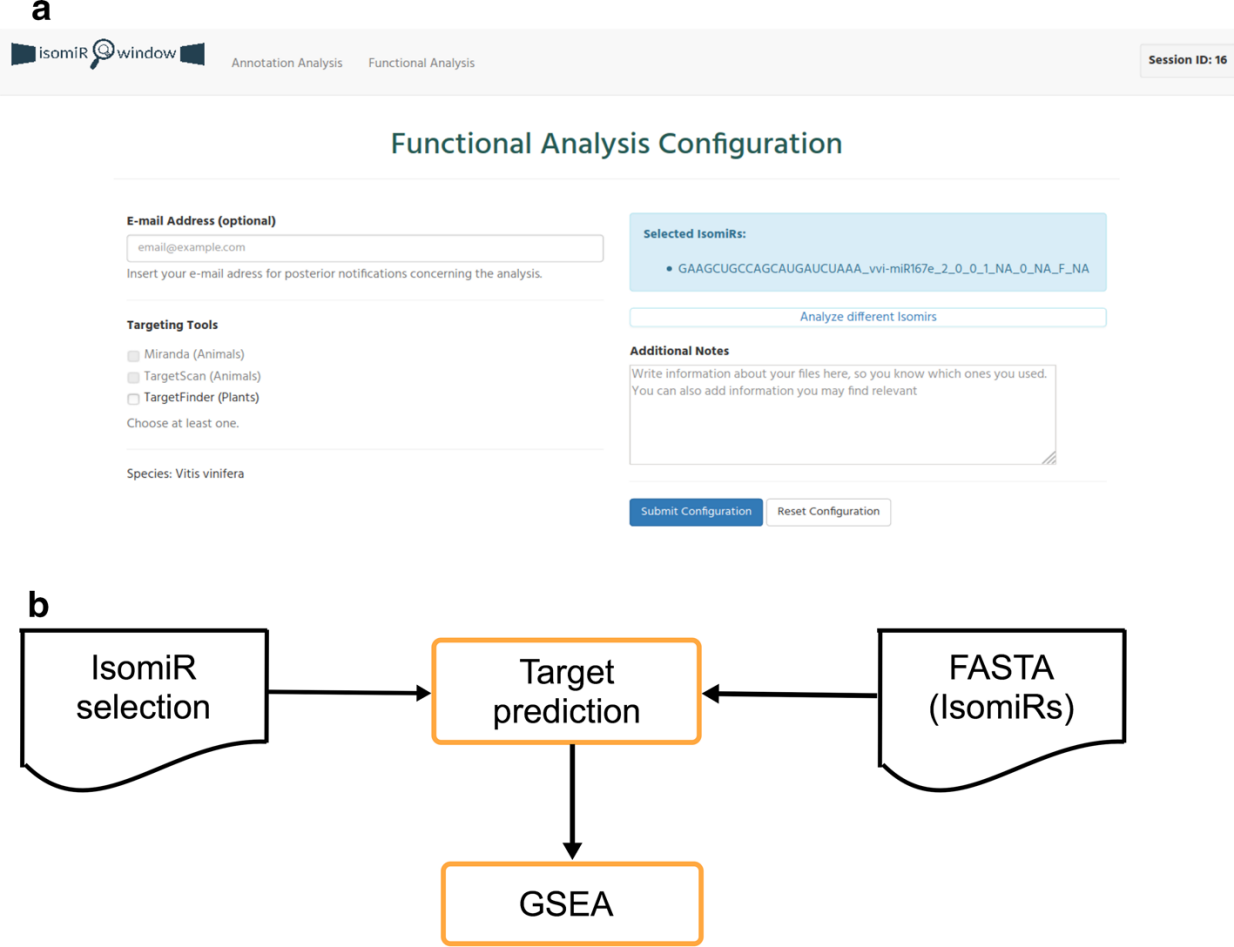
Functional analysis module. **a** Display of the configuration analysis form; **b** Module workflow

The Functional analysis module aims to identify isomiR targets, as well as to infer the pathways to which these are associated. This module is automatically launched by the IsomiR Window GUI after running the Annotation Analysis Module. Alternatively, it can be launched independently by the user. In this case, IsomiR Window directs the user to a form (Fig. 8a) where the user: (1) selects a FASTA file containing the sequences for target analysis as input; (2) selects the species; (3) selects the target prediction algorithm to be used and; (4) provides an email to receive updates regarding the analysis progress (optional). In case the user has selected to automatically run this module after the Annotation module, the user is redirected to a window for selecting the target prediction algorithm. IsomiR Window provides a selection of algorithms specific for animals [50, 51] and plants [52]. This page also displays the list of user-selected isomiRs during the preview of Annotation module results, allowing the user to change this selection. After this step, the user can submit the configuration form. The analysis parameters are automatically generated by the IsomiR Window GUI, which will execute the workflow of the IsomiR Window pipeline (Fig. 8b). The workflow starts by processing a FASTA file with the isomiR sequences to be analyzed, which was either uploaded by the user or generated automatically by the IsomiR Window GUI. This file is used as the input for the prediction of mRNA targets. In the case of animal genomes, the user may select the two available algorithms (Miranda and TargetScan). The output table will report the targets predicted in common by both algorithms. Nevertheless, the individual results generated by each prediction algorithm are available in the folder Functional Analysis/Results/gene_list*(Additional file 2: Fig. S2). After target prediction, the pipeline performs a gene set enrichment analysis, using R and the TopGO package [17].

### IsomiR Window identifies potential roles of isomiRs in BCC development

We have used IsomiR Window to investigate the effect of isomiRs in Basal Cell Carcinoma (BCCs) [36], a malignant tumor of follicular germinative cells (trichoblasts) that accounts for 75% of all skin cancers that is the most common malignant tumor in caucasian populations [53] The profile of sncRNAs was obtained for 15 patients in order to characterize the differences between Nodular (the most common and benign subtype) and Infiltrative (the most aggressive subtype) BCC. Data was preprocessed for adapter trimming and quality filtering. Filtered datasets presented on average 2 M reads and the most frequent read length was 22nt, in agreement with the expected read-length distribution for human miRNAs (Additional file 2: Fig. S3). At this stage, data occupied 5.7 Gb of disk space. After download and unpacking, the VM occupied ~ 200 Gb. The VM was configured to have 48 Gb of RAM, 5 CPUs and the VM disk (vdi) had a size of 38.50 Gb.

Filtered data was then used as an input to the IsomiR Window tool. The following parameters were set at the start of the Annotation Analysis module: (a) Experimental design—Unpaired samples; (b) Species—Homo sapiens; (c) Prediction of novel miRNAs—yes; (d) Experimental conditions to analyze when predicting novel miRNAs—both; (e) Minimum read stack for the prediction of novel miRNAs—10; (f) Use of annotation in the prediction of novel miRNAs—yes; (g) Include novel miRNAs in the identification of IsomiRs—yes; (h) Number of mismatches for read mapping—3; (i) Maximum number of genomic hits—5; (j) Significance level for DE—0.05. For the Functional Analysis module, both animal target prediction algorithms—Miranda and TargetScan—were selected.

**Fig. 9:**
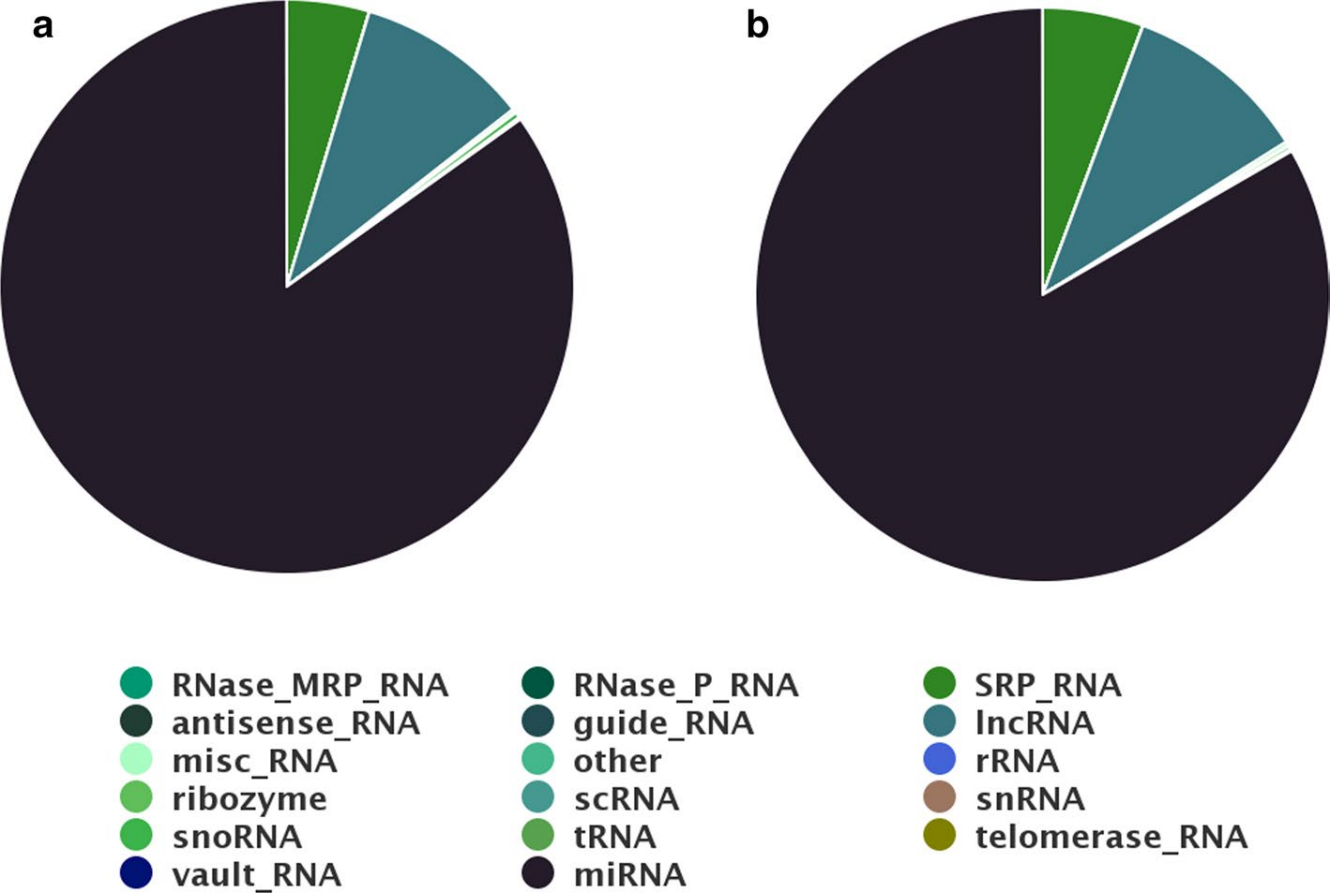
Proportion of ncRNA features in the BCCs datasets. Pie charts represent the mean of each feature estimated for each condition **a** Condition 1 (C1) Nodular BCCs; **b** Condition 2 (C2) Infiltrative BCCs

Results of IsomiR Window show that 94% of reads were successfully mapped to the reference genome across both conditions. Regarding the characterization of ncRNAs in the samples, Fig. 9 shows that more than 80% of the reads in both experimental conditions mapped to known miRNAs, with long non-coding RNAs (lncRNAs) and Signal Recognition Particle RNAs (SRP RNAs) representing the remaining 20%. Regarding the proportion of miRNAs that derive from each arm of the hairpin (and available feature of the IsomiR Window tool), most of the miRNAs expressed in BCCs were found to derive from the 5′ arm of the hairpin (Additional file 2: Fig. S4). As reported [36], the most expressed miRNAs in both types of BCC were hsa-miR-143-3p and hsa-miR-21-5p.

**Fig. 10:**
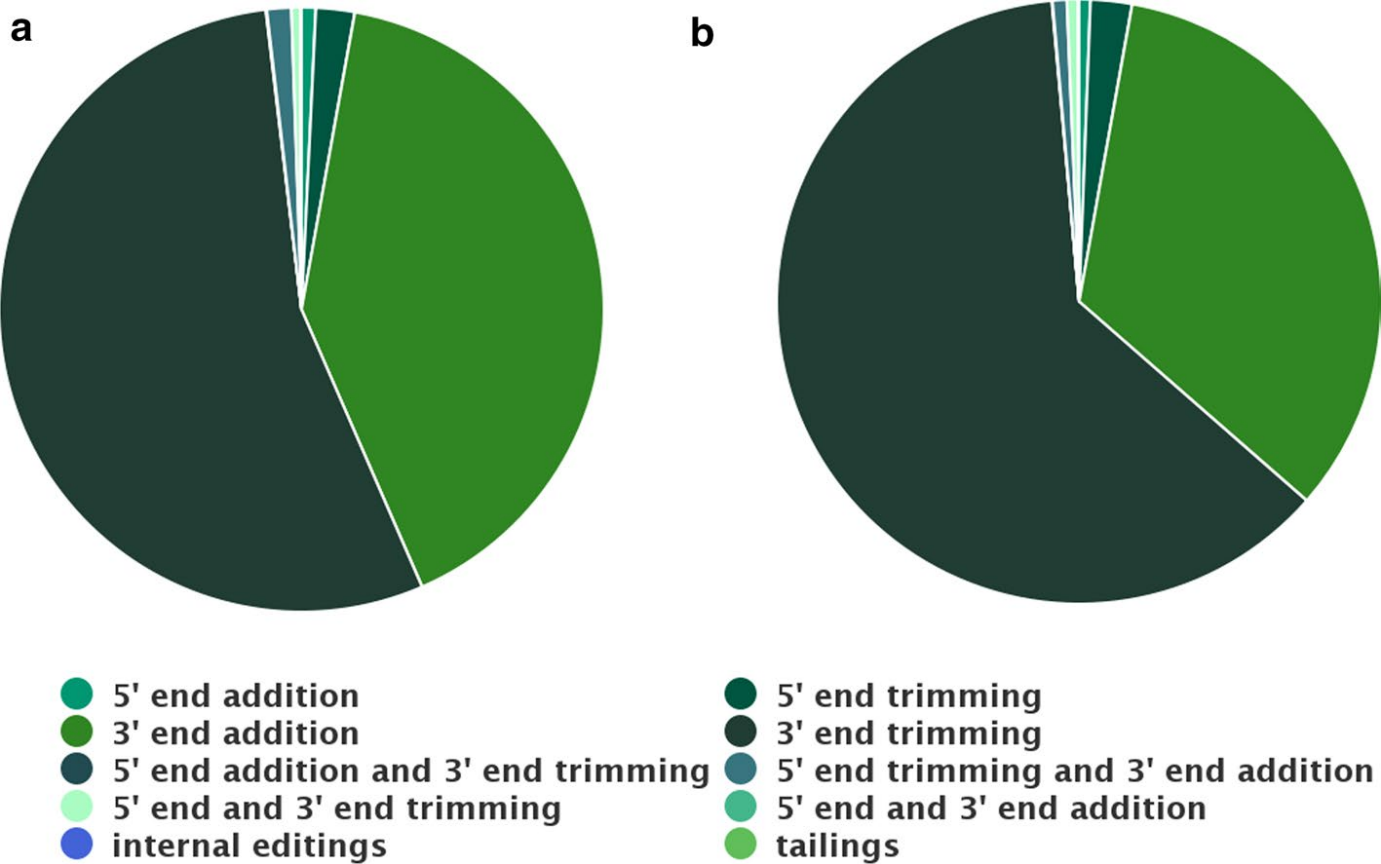
Proportion of different types of isomiRs identified in each experimental condition. Pie charts represent the mean of each feature estimated for each condition **a** Condition 1 (C1) Nodular BCCs; **b** Condition 2 (C2) Infiltrative BCCs

The analysis of isomiR species revealed that, for both conditions, most of the isomiRs differ from the respective canonical miR at the 3′ end, with 3′ end trimmings being the most frequent type of isomiR. These correspond to 54.6% and 62.1% of isomiRs in condition 1 and condition 2, respectively (Fig. 10). This result is in agreement with previous studies that have investigated the presence of isomiRs in different small-RNA-seq data [2, 54]. Although miRNAs with a common 5′ end but different 3′ ends are presumed to target the same mRNAs, they may differ in the extent of target repression or they might have different half-lives. Previous studies implicate 3′-to-5′ trimming as one major source of miRNA 3′ end heterogeneity [2, 54]. The IsomiR Window tool produces a bar plot that provides a detailed overview of the global profiles of miRNA variants in each experimental condition (Additional file 2: Fig. S5). Novel miRNA prediction analysis identified 155 previously unreported candidates for novel miRNAs, among which 79 intersect with other ncRNAs and 47 share the seed sequence with miRNAs known in other species. All these comparisons are automatically performed and shown in a table displayed by the tool (Additional file 1: Table S2). The output table includes a column identifying similar miRNAs existing in other species, as well as other ncRNAs annotated for the species and overlapping with the predicted novel miRNAs.

The last step of the Annotation analysis module is the differential expression (DE) of isomiR species. Although this analysis is only automated for isomiRs, the table of miRNA counts (with the sum of all miRNA variants of a given miRNA) is available in the folder Annotation Analysis/Results/miRNA_normalized_counts.txt (Additional file 2: Fig. S1) and the script for DE (Deseq.R) may be used to perfom DE analysis at the miRNA level.

**Fig. 11:**
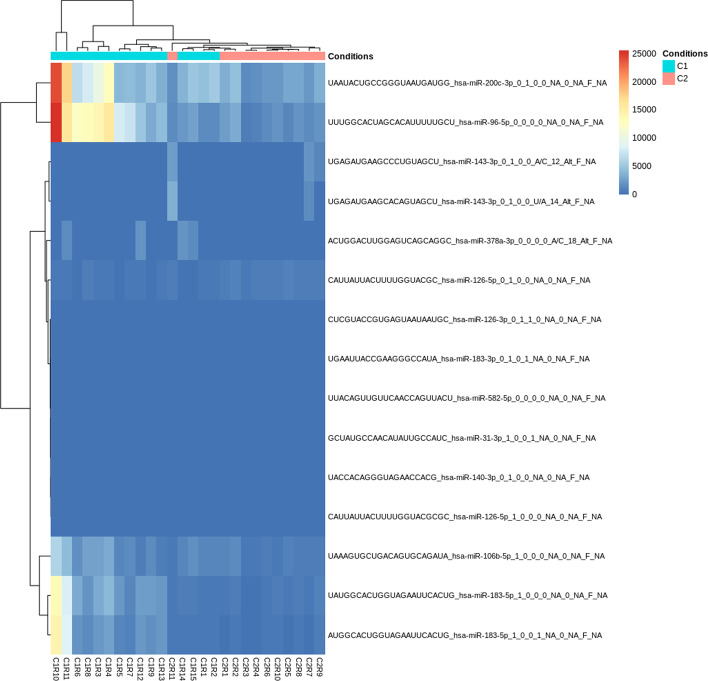
Heatmap displaying levels of expression of isomiRs that display significant differences between conditions. In case of the existence of a large number of differentially expressed (DE) isomiRs, the heatmap will display the top 15 DE. The plot shows that as previously reported at miRNA level [36], nodular BCCs display higher heterogeneicity in isomiR expression, as some samples cluster together with infiltrative BCCs

**Fig. 12:**
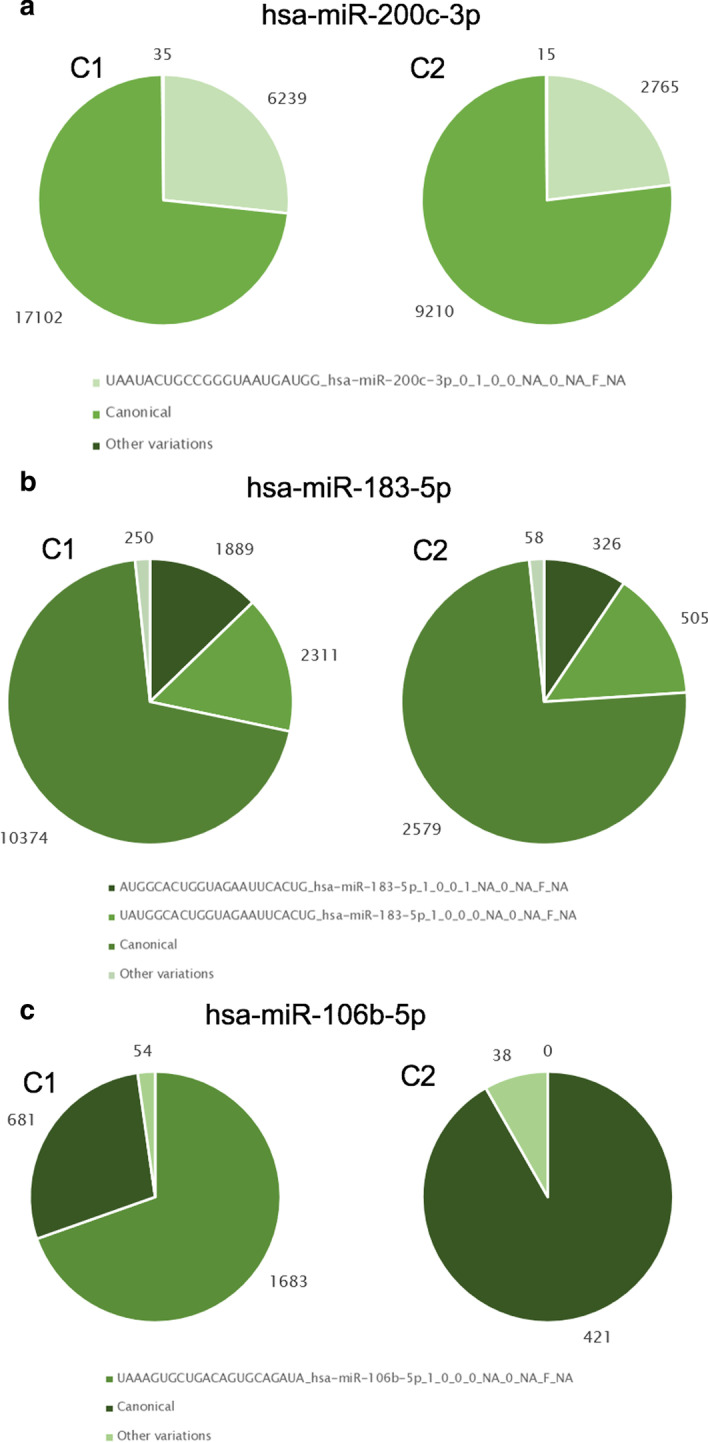
Proportion of normalized reads of canonical miRNAs and respective variants for DE IsomiRs. **a** hsa-miR-200c-3p; **b** hsa-miR-183-5p; **c** hsa-miR-106b-5p

A total of 12 isomiRs and 3 three canonical miRNAs were identified as being significantly differentially expressed, with the most contrasting species being upregulated in Nodular BCCs. This level of analysis was never performed before on this dataset. Since the read counts for each miRNA become distributed across the different miRNA variants, this analysis supports the characterization of a new level of complexity, as well as the precise identification of which miRNA variants are in fact altered across different conditions. Three of the identified miRNA variants contain fuzzy combinations that would not be detect by most of the isomiR detecting tools available (Table 1). Of the 12 identified variants, 5 corresponded to variants of canonical miRNAs identified in the original study [36] as significantly upregulated in nodular BCCs (Fig. 11). From these, we selected the three most contrasting variants to perform an analysis with the Functional module. Most of these isomiRs comprise a large proportion of the transcripts generated from the miRNA precursor, in comparison with the corresponding canonical form. Furthermore, differences in expression between the two types of BCCs were higher than three fold (Fig. 12). When analysing the potential functional impact of these isomiRs, we identified a specific set of targets (4% of the total) that comprised a group of genes displaying significant enrichment in biological processes critical for cancer development, namely, metabolism of RNA, DNA repair and signal transduction (Additional file 2: Fig. S6). One of the variants of miRNA 183-5p, whose canonical form was previously identified as being upregulated in Nodular BCCs, was found to be significantly upregulated in this type of tumours. IsomiR AUGGCACUGGUAGAAUUCACUG_hsa-miR-183-5p_1_0_0_1_NA_0_NA_F_NA is a 5′end variant of miR-183-5p that starts one nucleotide after the canonical form, thus presenting a different seed region. This is expected to affect mRNA targeting. In fact, target prediction results suggest that although this miRNA variant targets the same set of transcripts as its canonical form, it additionally presents 1756 isoform-exclusive mRNA targets that are significantly associated with disease pathways (Table 3).

**Table 3:** Top 25 pathways associated with specific mRNA targets of the isomiR AUGGCACUGGUAGAAUUCACUG_hsa-miR-183-5p_1_0_0_1_NA_0_NA_F_NA

Pathway name	Entities				Reactions	
	Found	Ratio	*p* value	FDR*	Found	Ratio
Defective EXT1 causes exostoses 1, TRFS2 and CHDS	5/16	7.87e-04	0.026	0.996	4/4	3.25e-04
Defective EXT2 causes exostoses 2	5/16	7.87e-04	0.026	0.996	4/4	3.25e-04
Defective B3GALT6 causes EDSP2 and SEMDJL1	5/21	0.001	0.067	0.996	1/1	8.12e-05
Defective B3GALT7 causes EDS, progeroid type	5/21	0.001	0.067	0.996	1/1	8.12e-05
Defective B3GAT3 causes JDSSDHD	5/22	0.001	0.079	0.996	1/1	8.12e-05
Negative regulation of TCF-dependent signaling by DVL-interacting proteins	2/5	2.46e-04	0.094	0.996	2/2	1.62e-04
Keratan sulfate biosynthesis	8/39	0.002	0.11	0.996	8/9	7.31e-04
NOTCH2 Activation and Transmission of Signal to the Nucleus	6/32	0.002	0.115	0.996	11/11	8.94e-04
HS-GAG biosynthesis	7/40	0.002	0.121	0.996	13/14	0.001
Interleukin-21 signaling	3/12	5.90e-04	0.127	0.996	5/5	4.06e-04
Phase 3- rapid repolarisation	3/12	5.90-e04	0.127	0.996	2/2	1.62e-04
O-linked glycosylation of mucins	11/73	0.004	0.136	0.996	17/17	0.001
Acyl chain remodelling of PS	6/34	0.002	0.14	0.996	4/8	6.50e-04
WNT ligand secretion is abrogated by PORCN inhibitor LGK974	1/2	9.84e-05	0.185	0.996	1/1	8.12e-05
Defective PMM2 causes PMM2-CDG (CDG-1a)	1/2	9.84e-05	0.185	0.996	1/1	8.12e-05
Defective SLC26A4 causes Pendred syndrome (PDS)	1/2	9.84e-05	0.185	0.996	1/1	8.12e-05
Acyl chain remodelling of PI	5/30	0.001	0.198	0.996	3/6	4.87e-04
RNF mutatnts show enhanced WNT signaling and proliferation	2/8	3.93e-04	0.199	0.996	1/1	8.12e-05
Mitochondrial calcium ion transport	5/31	0.002	0.216	0.996	5/10	8.12e-04
Retinoid metabolismo and transport	17/107	0.005	0.216	0.996	10/28	0.002
IRAK1 recruits IKK complex	3/16	7.87e-04	0.227	0.996	4/4	3.25e-04
IRAK1 recruits IKK complex upon TLR7/8 or 9 stimulation	3/16	7.87e-04	0.227	0.996	4/4	3.25e-04
Defective B4GALT1 causes B4GALT1-CDG (CDG-2d)	2/9	4.43e-04	0.236	0.996	3/3	2.44e-04
Erythropoietin activates STAT5	2/9	4.43e-04	0.236	0.996	3/3	2.44e-04
STAT5 activation	2/9	4.43e-04	0.236	0.996	3/3	2.44e-04

In the analysis directories, original result files are available that enable further exploration of other relevant aspects, such as (1) the presence of other ncRNAs, since these are also quantified for each sample of each condition; (2) miRNA level quantification of expression; and (3) lists of predicted mRNA targets per isomiR or miRNA.

Regarding the usage of resources, SWAP utilization is zero throughout all the workflow, showing that memory requirements were sufficient. The process required 7 Gb of memory. For the moment the tool is not parallelizing (Additional file 2: Fig. S7) processes. The implementation of parallel process instructions in the Laravel-based workflow manager can be used for improving runtime and is under consideration as a future development of the pipeline in the short term.In summary, IsomiR Window supports a detailed exploration of the complexity of miRNA biology, further enabling a straight-forward analysis of small-RNA-seq data.

## Availability and requirements

Project name: IsomiR Window

Project home page: https://isomir.fc.ul.pt/; https://github.com/andreiaamaral/IsomiR-Window

Operating system(s): Platform independent

Programming language: Laravel framework, PHP, HTML, Java, MySQL, PERL

Other requirements: not applicable, the tool is deployed in a Virtual Machine with all requirements set

License: GNU General Public License v3.0

## Supplementary Information

**Additional file 1.** Supplementary Tables.

**Additional file 2.** Supplementary Figures.

## Data Availability

All data analysed during this study are included in this published article.

## Abbreviations

NGS: Next generation sequencing
VM: Virtual machine
KB: Knowledge bases

## References

[1] Bartel DP, Lee R, Feinbaum R (2004). MicroRNAs: genomics biogenesis, mechanism, and function genomics: the miRNA genes. Cell.

[2] Neilsen CT, Goodall GJ, Bracken CP (2012). IsomiRs—the overlooked repertoire in the dynamic microRNAome. Trends Genet.

[3] Esteller M (2011). Non-coding RNAs in human disease. Nat Rev Genet.

[4] Wu X, Zeng R, Wu S, Zhong J, Yang L, Xu J (2015). Comprehensive expression analysis of miRNA in breast cancer at the miRNA and isomiR levels. Gene.

[5] Boele J, Persson H, Shin JW, Ishizu Y, Newie IS, Sokilde R (2014). PAPD5-mediated 3’ adenylation and subsequent degradation of miR-21 is disrupted in proliferative disease. Proc Natl Acad Sci.

[6] Dodt M, Roehr J, Ahmed R, Dieterich C (2012). FLEXBAR—flexible barcode and adapter processing for next-generation sequencing platforms. Biology (Basel).

[7] Martin M (2011). Cutadapt removes adapter sequences from high-throughput sequencing reads. EMBnet J.

[8] Andrews S. FastQC A Quality Control tool for High Throughput Sequence Data. 2010. http://www.bioinformatics.babraham.ac.uk/projects/fastqc/. Accessed 1 Dec 2013.

[9] Li H, Durbin R (2009). Fast and accurate short read alignment with Burrows-Wheeler transform. Bioinformatics.

[10] Langmead B. Aligning short sequencing reads with Bowtie. Curr Protoc Bioinformatics. 2010;CHAPTER:Unit-11.7. doi:10.1002/0471250953.bi1107s32. 10.1002/0471250953.bi1107s32 PMC3010897 21154709

[11] Anders S, Huber W (2010). Differential expression analysis for sequence count data. Genome Biol.

[12] Friedman RC, Farh KKH, Burge CB, Bartel DP (2009). Most mammalian mRNAs are conserved targets of microRNAs. Genome Res.

[13] Enright AJ, John B, Gaul U, Tuschl T, Sander C, Marks DS (2003). MicroRNA targets in Drosophila. Genome Biol.

[14] Kertesz M, Iovino N, Unnerstall U, Gaul U, Segal E (2007). The role of site accessibility in microRNA target recognition. Nat Genet.

[15] Friedländer MR, Mackowiak SD, Li N, Chen W, Rajewsky N (2012). miRDeep2 accurately identifies known and hundreds of novel microRNA genes in seven animal clades. Nucleic Acids Res.

[16] Falcon S, Gentleman R (2007). Using GOstats to test gene lists for GO term association. Bioinformatics.

[17] Alexa A, Rahnenfuhrer J. topGO: Enrichment Analysis for Gene Ontology. R package version 2.32.0. 2016.

[18] Griffiths-Jones S, Grocock RJ, van Dongen S, Bateman A, Enright AJ (2006). miRBase: microRNA sequences, targets and gene nomenclature. Nucleic Acids Res.

[19] Flicek P, Amode MR, Barrell D, Beal K, Billis K, Brent S (2014). Ensembl 2014. Nucleic Acids Res.

[20] Consortium TRna (2017). RNAcentral: a comprehensive database of non-coding RNA sequences. Nucleic Acids Res..

[21] Kaushik A, Saraf S, Mukherjee SK, Gupta D (2015). miRMOD: a tool for identification and analysis of 5′ and 3′ miRNA modifications in Next Generation Sequencing small RNA data. PeerJ.

[22] Zhang Y, Xu B, Yang Y, Ban R, Zhang H, Jiang X (2012). Cpss: A computational platform for the analysis of small rna deep sequencing data. Bioinformatics.

[23] Wan C, Gao J, Zhang H, Jiang X, Zang Q, Ban R (2017). CPSS 2.0: a computational platform update for the analysis of small RNA sequencing data. Bioinformatics.

[24] Zhang Y, Zang Q, Zhang H, Ban R, Yang Y, Iqbal F (2016). DeAnnIso: a tool for online detection and annotation of isomiRs from small RNA sequencing data. Nucleic Acids Res.

[25] Sablok G, Milev I, Minkov G, Minkov I, Varotto C, Yahubyan G (2013). IsomiRex: Web-based identification of microRNAs, isomiR variations and differential expression using next-generation sequencing datasets. FEBS Lett.

[26] Guo L, Yu J, Liang T, Zou Q (2016). MIR-isomiRExp: a web-server for the analysis of expression of miRNA at the miRNA/isomiR levels. Sci Rep..

[27] Humphreys DT, Suter CM. MiRspring: a compact standalone research tool for analyzing miRNA-seq data. Nucleic Acids Res. 2013;41:e147:1–8. 10.1093/nar/gkt485 PMC3753622 23775795

[28] Muller H, Marzi MJ, Nicassio F (2014). IsomiRage: from functional classification to differential expression of miRNA isoforms. Front Bioeng Biotechnol.

[29] De Oliveira LFV, Christoff AP, Margis R (2013). isomiRID: a framework to identify microRNA isoforms. Bioinformatics.

[30] Vitsios DM, Enright AJ (2015). Chimira: analysis of small RNA sequencing data and microRNA modifications. Bioinformatics.

[31] Rueda A, Barturen G, Lebrón R, Gómez-Martín C, Alganza Á, Oliver JL (2015). SRNAtoolbox: an integrated collection of small RNA research tools. Nucleic Acids Res.

[32] Aparicio-Puerta E, Lebrón R, Rueda A, Gómez-Martín C, Giannoukakos S, Jaspez D (2019). sRNAbench and sRNAtoolbox 2019: intuitive fast small RNA profiling and differential expression. Nucleic Acids Res.

[33] Kesharwani RK, Chiesa M, Bellazzi R, Colombo GI (2019). CBS-miRSeq: a comprehensive tool for accurate and extensive analyses of microRNA-sequencing data. Comput Biol Med.

[34] Sun Z, Evans J, Bhagwate A, Middha S, Bockol M, Yan H (2014). CAP-miRSeq: A comprehensive analysis pipeline for microRNA sequencing data. BMC Genomics.

[35] Mohorianu I, Stocks MB, Applegate CS, Folkes L, Moulton V (2017). The UEA small RNA Workbench: a suite of computational tools for small RNAanalysis. Methods Mol Biol..

[36] Heffelfinger C, Ouyang Z, Engberg A, Leffell DJ, Hanlon AM, Gordon PB (2012). Correlation of global microRNA expression with basal cell carcinoma subtype. G3 Genes Genomes Genet.

[37] R Development Core Team R. R: a language and environment for statistical computing. R Foundation for Statistical Computing. 2011;1 2.11.1:409. doi:10.1007/978-3-540-74686-7.

[38] Taylor O. Laravel The PHP Framework for Web Artisans.

[39] Huang W, Li L, Myers JR, Marth GTART (2012). A next-generation sequencing read simulator. Bioinformatics.

[40] Griffiths-Jones S (2006). miRBase: microRNA sequences, targets and gene nomenclature. Nucleic Acids Res..

[41] Schmieder R, Edwards R (2011). Quality control and preprocessing of metagenomic datasets. Bioinformatics.

[42] Ziemann M, Kaspi A, El-Osta A (2016). Evaluation of microRNA alignment techniques. RNA.

[43] Anders S. Htseq: Analysing high-throughput sequencing data with python. 2010. http://www-huber.embl.de/users/anders/HTSeq/doc/count.html. 10.1093/bioinformatics/btac166 PMC9113351 35561197

[44] Kuang Z, Wang Y, Li L, Yang X (2019). miRDeep-P2: accurate and fast analysis of the microRNA transcriptome in plants. Bioinformatics.

[45] Li H, Handsaker B, Wysoker A, Fennell T, Ruan J, Homer N (2009). The sequence alignment/map format and SAMtools. Bioinformatics.

[46] Narasimhan V, Danecek P, Scally A, Xue Y, Tyler-Smith C, Durbin R (2016). BCFtools/RoH: a hidden Markov model approach for detecting autozygosity from next-generation sequencing data. Bioinformatics.

[47] DePristo MA, Banks E, Poplin R, Garimella K, Maguire J, Hartl C (2011). A framework for variation discovery and genotyping using next-generation DNA sequencing data. Nat Genet.

[48] Sherry ST (2001). dbSNP: the NCBI database of genetic variation. Nucleic Acids Res.

[49] Savva YA, Rieder LE, Reenan RA (2012). The ADAR protein family. Genome Biol.

[50] Enright AJ, John B, Gaul U, Tuschl T, Sander C, Marks DS (2003). MicroRNA targets in Drosophila.

[51] Lewis BP, Burge CB, Bartel DP (2005). Conserved seed pairing, often flanked by adenosines, indicates that thousands of human genes are microRNA targets. Cell.

[52] Fahlgren N, Howell M, Kasschau K (2008). High-throughput sequencing of arabidopsis MicroRNAs: Evidence for frequent birth and death of MIRNA genes. Chemtracts.

[53] Dessinioti C, Antoniou C, Katsambas A, Stratigos AJ (2010). Basal cell carcinoma: what’s new under the sun. Photochem Photobiol.

[54] Ameres SL, Zamore PD (2013). Diversifying microRNA sequence and function. Nat Rev Mol Cell Biol.

