# Supplementary material for: IsomiR_Window: a system for analyzing small-RNA-seq data in an integrative and user-friendly manner
Source: BMC Bioinformatics. 2021 Feb 1;22:37. doi: 10.1186/s12859-021-03955-6 (PMC7852101; doi:10.1186/s12859-021-03955-6)

**Table S1** - Third party software made available and used in the IsomiR Window tool.

| Tool | Version | Tool | Version |
| --- | --- | --- | --- |
| BEDTools | 2.26.0 | miRDeep2 | 2.0.1.2 |
| Bowtie | 1.2.2 | miRDP2 | 1.1.2 |
| DESeq2 | 1.24.0 | SAMtools | 1.5 |
| GATK | 3.8-1-0 | TargetFinder | Aug 2016 |
| HTSeq | 0.6.1p1 | TargetScan | 7.0 |
| miRanda | v3.3a | topGO | 2.26.0 |

**Table S2 –** Prediction of novel miRNAs using miRDeep2. The table displays the prediction of miRDeep2, the tool displays results that showed a positive prediction of secondary structure by Randfold as well as it displays possible overlaps with other annotated ncRNAs.


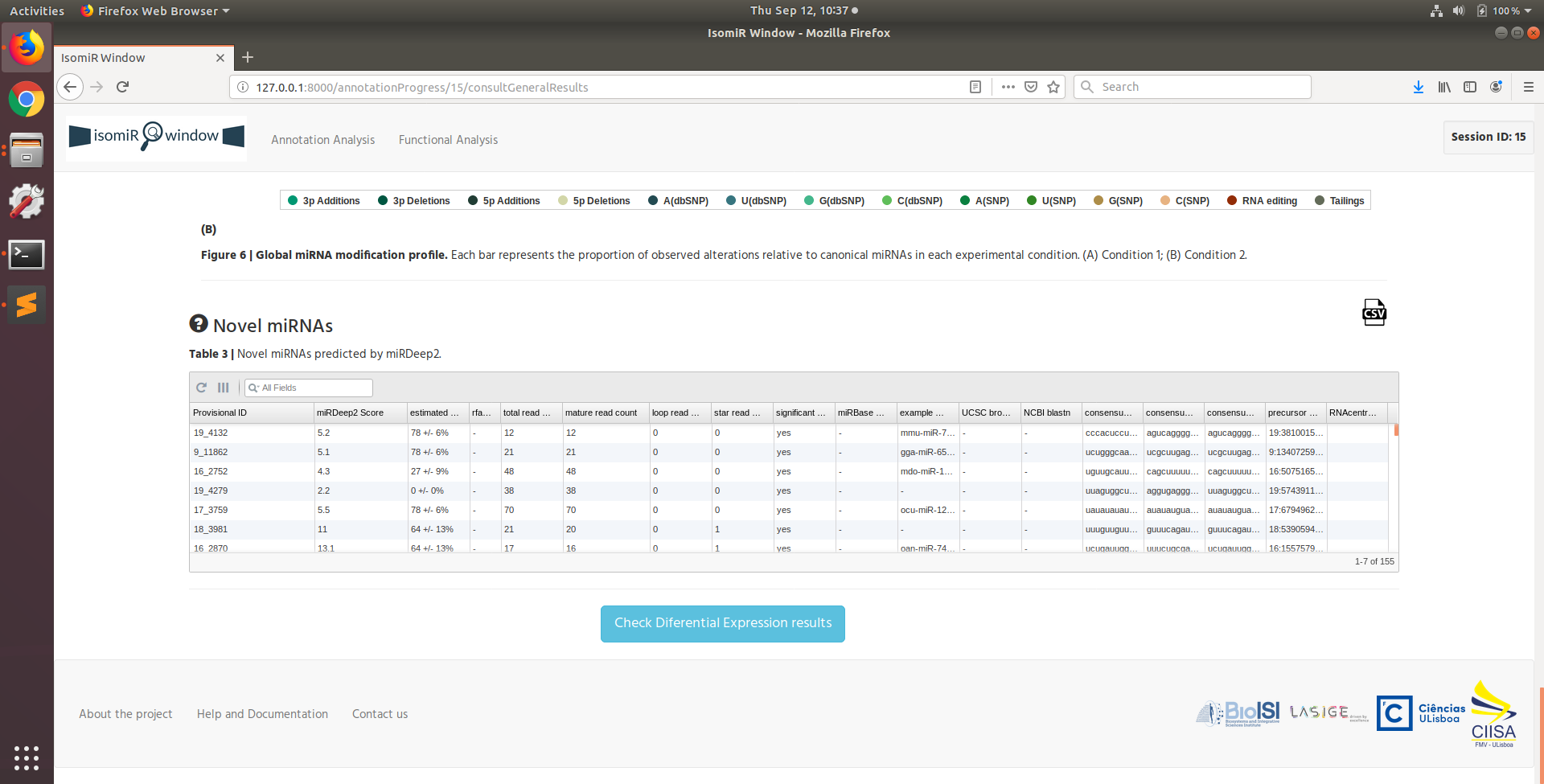

Supplement: Supplementary file 1 — Additional file 1. Supplementary Tables. [file 12859_2021_3955_MOESM1_ESM.docx]
