# Supplementary material for: IsomiR_Window: a system for analyzing small-RNA-seq data in an integrative and user-friendly manner
Source: BMC Bioinformatics. 2021 Feb 1;22:37. doi: 10.1186/s12859-021-03955-6 (PMC7852101; doi:10.1186/s12859-021-03955-6)
Supplement: Supplementary file 2 — Additional file 2. Supplementary Figures. [file 12859_2021_3955_MOESM2_ESM.docx]

Supplemental Figures


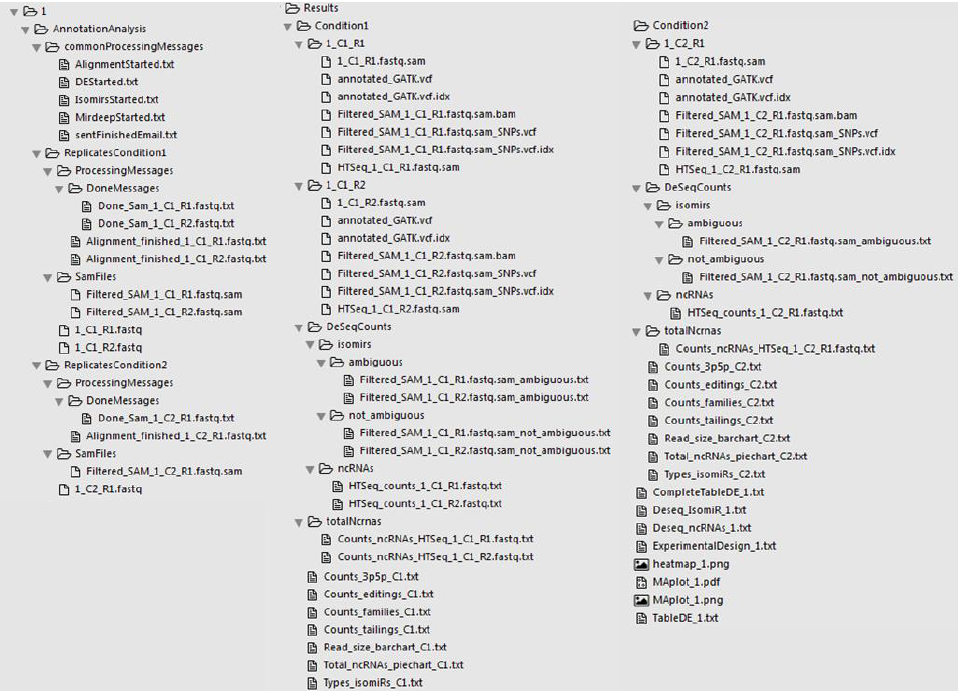


Figure S1- File organization of IsomiR Window of Annotation analysis module.


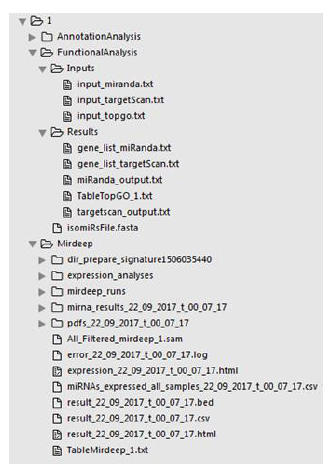


Figure S2- File organization of IsomiR Window of Functional analysis module.


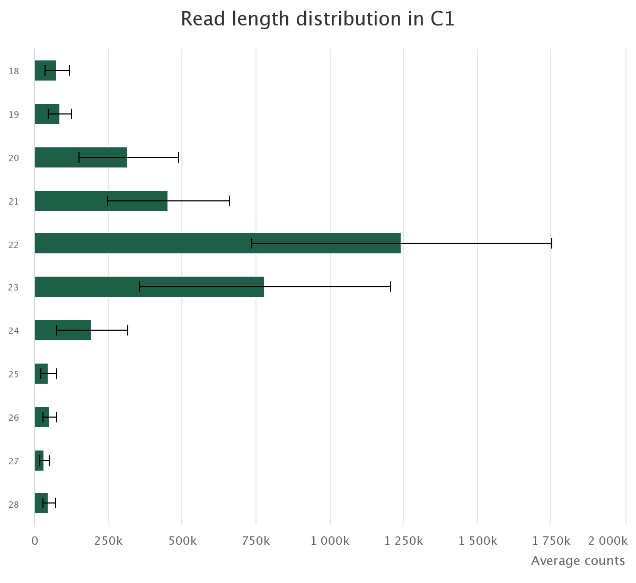

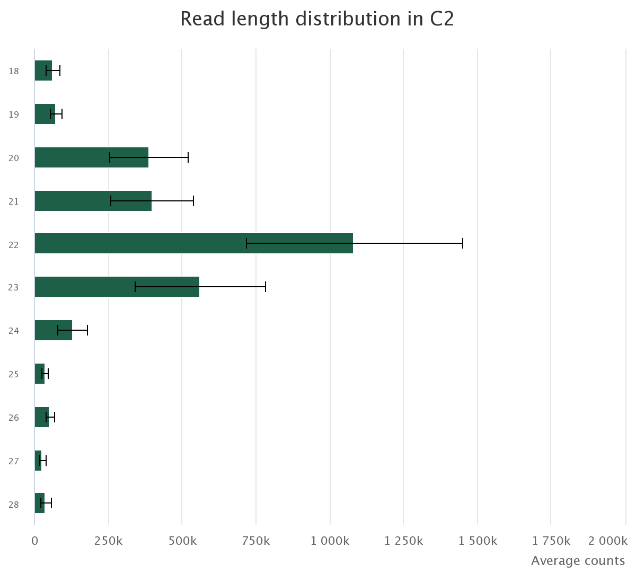


**Figure S3 -** Average read length distribution in each experimental condition. C1- Nodular BCCs; C2- Infiltrative BCCs. Bars represent the mean across biological replicates of each of the biological conditions. Vertical black lines represent the estimated standard deviation.


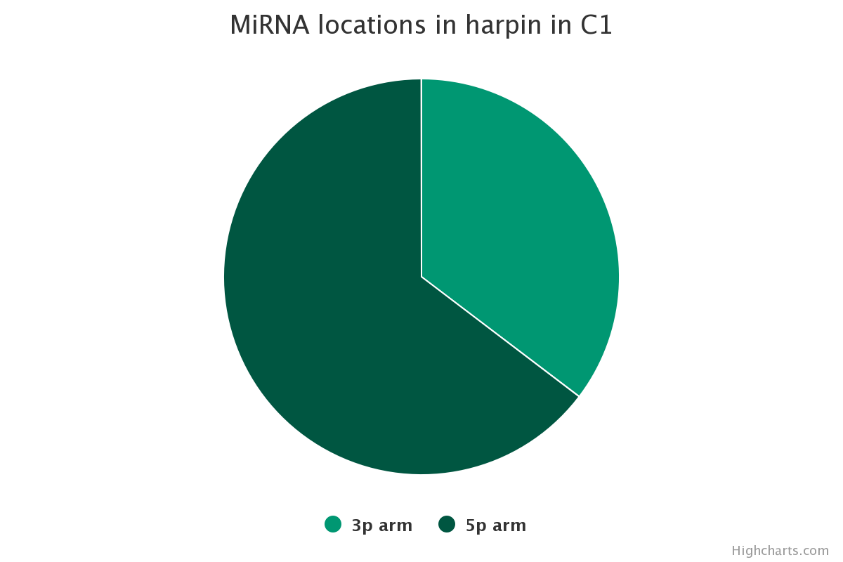

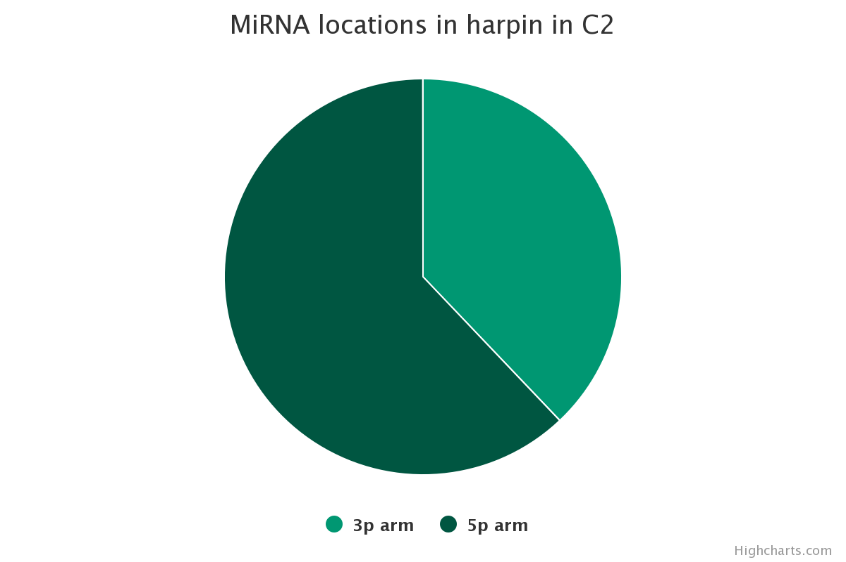


**Fig. S4 -** Proportion of miRNAs that are originated in the 3p arm or in the 5p arm of the miRNA hairpin in each experimental condition. C1- Nodular BCCs; C2- Infiltrative BCCs. The pie chart represent the estimated mean of each feature across the biological replicates.


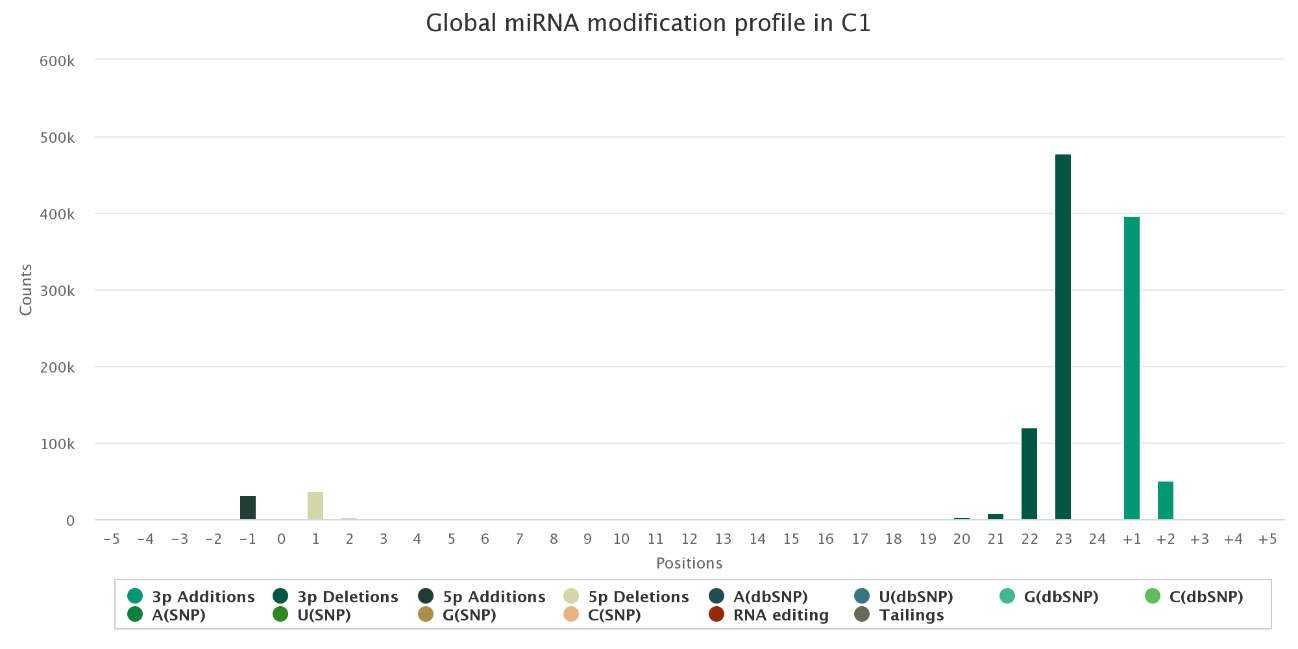


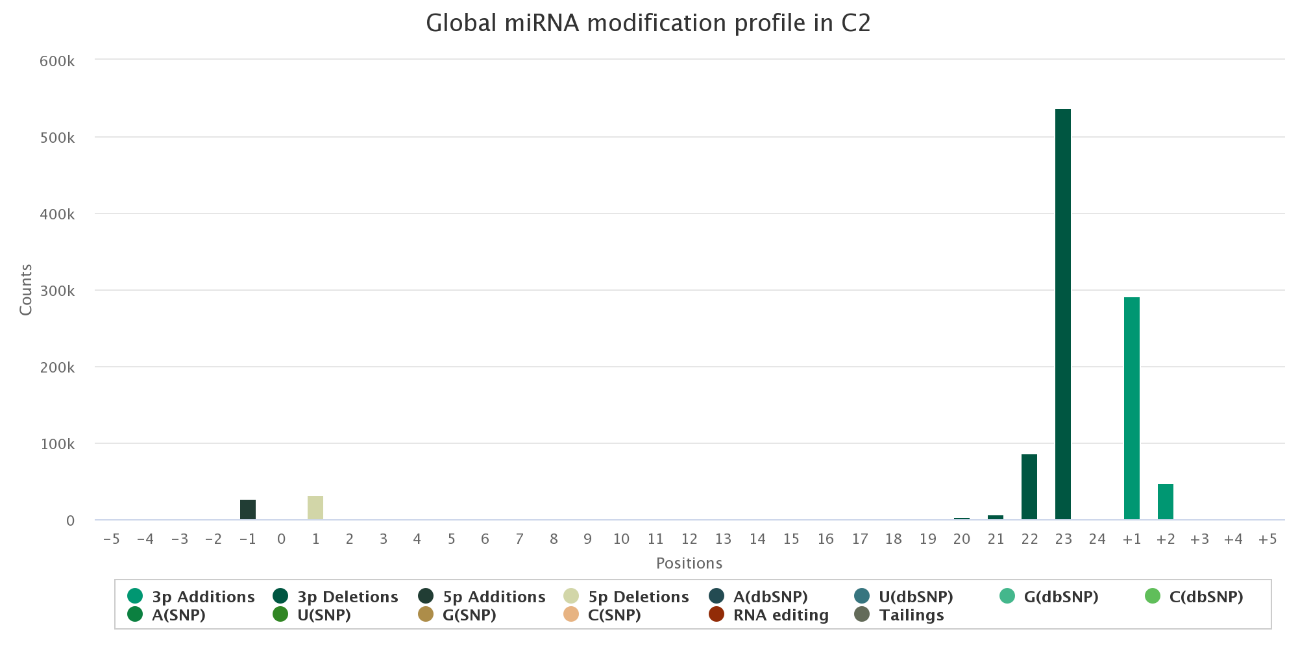


**Fig. S5**  - Global miRNA modification profile. C1- Nodular BCCs; C2- Infiltrative BCCs. Each bar represents the cumulative normalized counts. RNA editing correspond to potential ADAR editing’s.


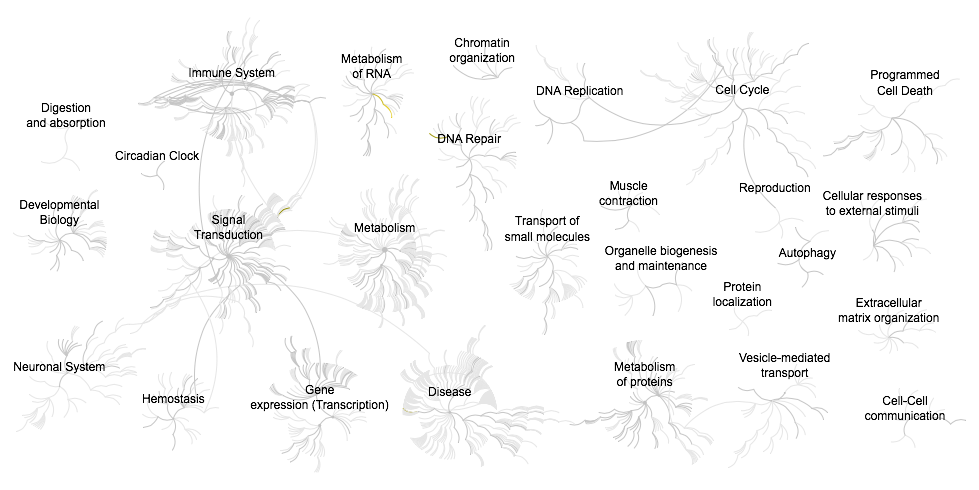


**Fig. S6 -** Genome-wide overview of pathway analysis of mRNA targets potentially targeted uniquely by differentially expressed IsomiRs.

(A)


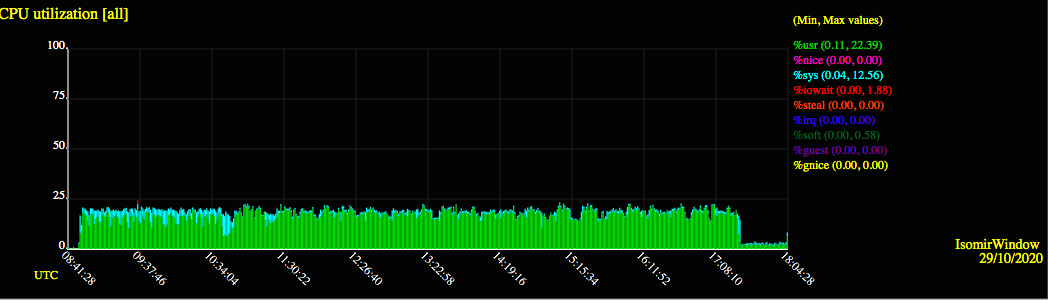


(B)


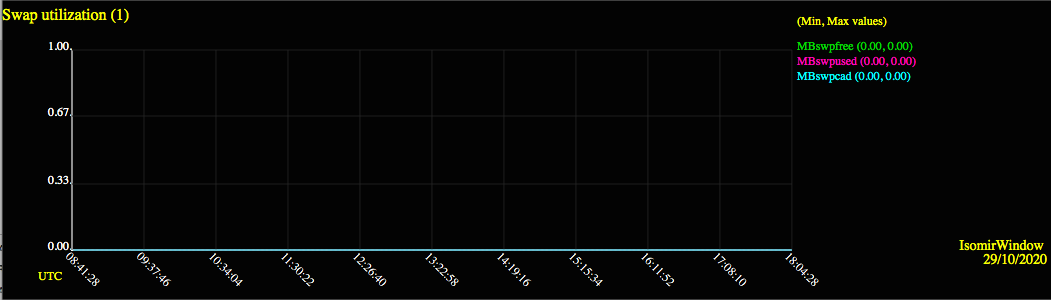


**Fig. S7**- IsomiR Window resource usage during demonstration analysis.
